# Melting Temperature Hidden Behind Liquid–Liquid Phase Transition in Glycerol

**DOI:** 10.1021/acs.jpcb.4c04552

**Published:** 2025-01-23

**Authors:** Szymon Starzonek, Jakub Kalabiński, Aleksandra Drozd-Rzoska, Sylwester J. Rzoska, Aleš Iglič

**Affiliations:** † Institute of Theoretical Physics and Mark Kac Center for Complex Systems Research, Jagiellonian University, Kraków 30-348, Poland; ‡ Institute of High Pressure Physics of the Polish Academy of Sciences, Warsaw 01-142, Poland; § Laboratory of Physics, Faculty of Electrical Engineering, University of Ljubljana, Ljubljana 1000, Slovenia

## Abstract

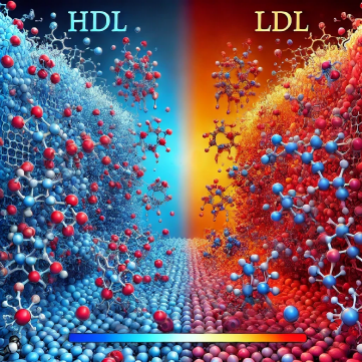

Liquid–liquid phase transitions play a pivotal role in various scientific disciplines and technological applications, ranging from biology to materials science and geophysics. Understanding the behavior of materials undergoing these transitions provides valuable insights into complex systems and their dynamic properties. This review explores the implications of liquid–liquid phase transitions, particularly focusing on the transition between low-density liquid (LDL) and high-density liquid (HDL) phases. We investigate the thermodynamic, structural, and mechanistic aspects of these transitions, emphasizing their relevance in diverse fields. The creation of dynamic heterogeneities and critical fluctuations during liquid–liquid phase transitions is discussed, highlighting their role in shaping the phase behavior and dynamics of complex fluids. Experimental observations, including the use of dielectric spectroscopy and nonlinear methods, shed light on the intricate nature of these transitions. Our findings suggest a connection between liquid–liquid phase transitions and critical phenomena, with implications for understanding the supercooled state and phase behavior of hydrogen-bonded liquids such as glycerol. Overall, this review underscores the importance of interdisciplinary approaches in unraveling the complexities of liquid–liquid phase behavior and addressing fundamental questions.

## Introduction

Liquid–liquid (LL) phase transitions have implications across various scientific disciplines and technological applications.^1−5^ In biology, for example, understanding the behavior of intracellular water can provide insights into cellular processes and biomolecular interactions. In materials science, the design of new functional materials often relies on controlling phase transitions to achieve desired properties.^5,6^ Furthermore, the study of LL phase transitions in geophysical systems, such as Earth’s mantle, has implications for understanding planetary dynamics and evolution.^7,8^

Melting is a first-order transition from a solid to a liquid phase, taking place at a characteristic temperature . According to phenomenological thermodynamics, this process is characterized by the reduction of the Gibbs free energy (Δ*G*) to zero and nonzero changes in enthalpy (Δ*H*) and entropy (Δ*S*).^9^ Another important criterion of melting is that the Gibbs free energy of the liquid is lower than that of the solid.^10^ On the other hand, glass-forming liquids and melted metals exhibit a lambda-like transition between the liquid and supercooled states.^3,11^ Refs (12) and (13) indicate that such behavior can also be found in water and its metastable state.

Liquid–liquid phase transitions have attracted significant attention due to their relevance in understanding the behavior of diverse materials ranging from simple liquids to complex biological systems. Of particular interest are transitions between low-density liquid (LDL) and high-density liquid (HDL) phases, which have been observed in various substances, including water, silicon, and silica.^4,9,11−13^ These LL phase transitions occur under specific conditions of temperature, pressure, and composition, and elucidating their underlying mechanisms is crucial for understanding the properties of materials in different states.

LL phase transitions are intimately connected to the creation of dynamic heterogeneities and critical fluctuations, which are crucial phenomena in understanding the behavior of complex fluids.^14−17^ These phenomena arise due to the inherent structural and dynamical complexities present in systems undergoing LL phase transitions.

Several mechanisms have been proposed to explain the driving forces behind LL phase transitions.^1,2,4,9,12,13,17^ Among these are changes in hydrogen bonding, structural rearrangements, and percolation phenomena. In water,^1,17−19^ for instance, the competition between hydrogen bonding networks leads to the formation of LDL and HDL phases under specific conditions. In other systems, such as colloidal suspensions,^20,21^ changes in particle interactions and crowding effects play a significant role in driving phase transitions.

Barrat^22^ and Glotzer et al.^23^ introduced a critical-like description for nonlinear susceptibility (χ_4_) related to a four-point correlation density function in hard-sphere liquids. They showed that, based on the mean-field approach, the critical exponent describing the nonlinear susceptibility is  upon approaching from below and above the critical temperature *T*_c_. During LL phase transitions, regions of differing densities and local structures emerge as the system traverses the coexistence region between the LDL and HDL phases. These heterogeneous regions, referred to as domains, exhibit distinct dynamic behaviors, including variations in diffusion rates, viscosity, and molecular arrangements.^22,23^ Near the critical point, the correlation length diverges, leading to the emergence of large-scale density fluctuations. These fluctuations manifest as regions of enhanced density or compositional fluctuations, giving rise to heterogeneous structures within the fluid. In the context of LL phase transitions, critical fluctuations play a crucial role in promoting the formation of dynamic heterogeneities by driving the system toward criticality, where fluctuations become long-ranged and exhibit scale invariance.^17,24,25^

Furthermore, the presence of dynamic heterogeneities and critical fluctuations influences the kinetics of phase separation, affecting the dynamics of the nucleation, growth, and coarsening processes. Nonequilibrium effects, such as spinodal decomposition and droplet coalescence, are governed by the spatial and temporal correlations arising from dynamic heterogeneities and critical fluctuations. Understanding the role of these phenomena in controlling phase transitions is essential for predicting and manipulating the phase behavior of complex fluids under different thermodynamic conditions.

## Experimental Section

In soft matter systems, like liquid crystals, supercooled liquids, etc., the broadband dielectric spectroscopy is very helpful and commonly used.^25−32^ It is based on the electrical measurements of complex dielectric permittivity, , as a function of temperature and pressure.^33^ By taking a value of the real part  at a plateau region in the spectrum, which for soft matter systems occurs for  kHz, one may define the dielectric constant, .^27,28^ The temperature (or pressure) change of the value of  describes the orientation of dipole moments of molecules, which is commonly used to study phase transitions.^27−32^ Since broadband dielectric spectroscopy is based on a two-point correlation function,^26,34^ which gives only a macroscopic portrayal, other sophisticated methods are the nonlinear dielectric effect (NDE) and third-harmonics analysis.^26−28,31,34^ These approaches allow the separation of dielectric response of molecules enclosed in a cluster from the background.^26−32^ Rzoska et al.^28,29^ proposed a critical-like description of the temperature dependency of the nonlinear dielectric effect *ε*_NDE_ for complex liquids. Comparing the above with^22,23^ it leads to the following relation:
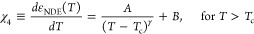
1
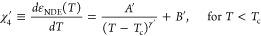
2where *T*_c_ denotes the critical temperature, γ is the critical exponent for the three-dimensional Ising (*d* = 3, *n* = 1) universality class,^23,27^ and *A* and *B* are the constants.

The pure glycerol (99.9%) was purchased from Sigma-Aldrich and was heated under vacuum to remove the rest of the bonded water. The concentration of water after heating was less than 0.05% (Karl Fischer titration and DSC). The capacitor, with a diameter of 2r = 20 mm and a gap *d* = 0.2 mm, was filled with glycerol at a high temperature (above 373 K) and cooled to room temperature. Experiments were carried out by the use of Novocontrol Alpha-A Impedance Analyzer with wide ranges of frequency (10^7^–10^–2^ Hz) and temperature (305–280 K) with a step  K and a stability better than  K. Nonlinear parameters such as third harmonics  and nonlinear dielectric effect (*ε*_NDE_) were measured using the Novocontrol HV/Trek Amplifier setup. The obtained data were analyzed by the use of derivative methods described here.^28−31,33−35^

To calculate the DC conductivity (σ_DC_) from dielectric spectra, one may use the frequency-dependent complex conductivity (), which is related to the complex permittivity: . In the dielectric spectrum,  typically shows a plateau at low frequencies corresponding to σ_DC_. Therefore, the value of  in this low-frequency plateau is the DC conductivity. Alternatively, if the dielectric loss  is dominated by conductive contributions at low frequencies, one may estimate σ_DC_ using .

## Results and Discussion

**Figure 1:**
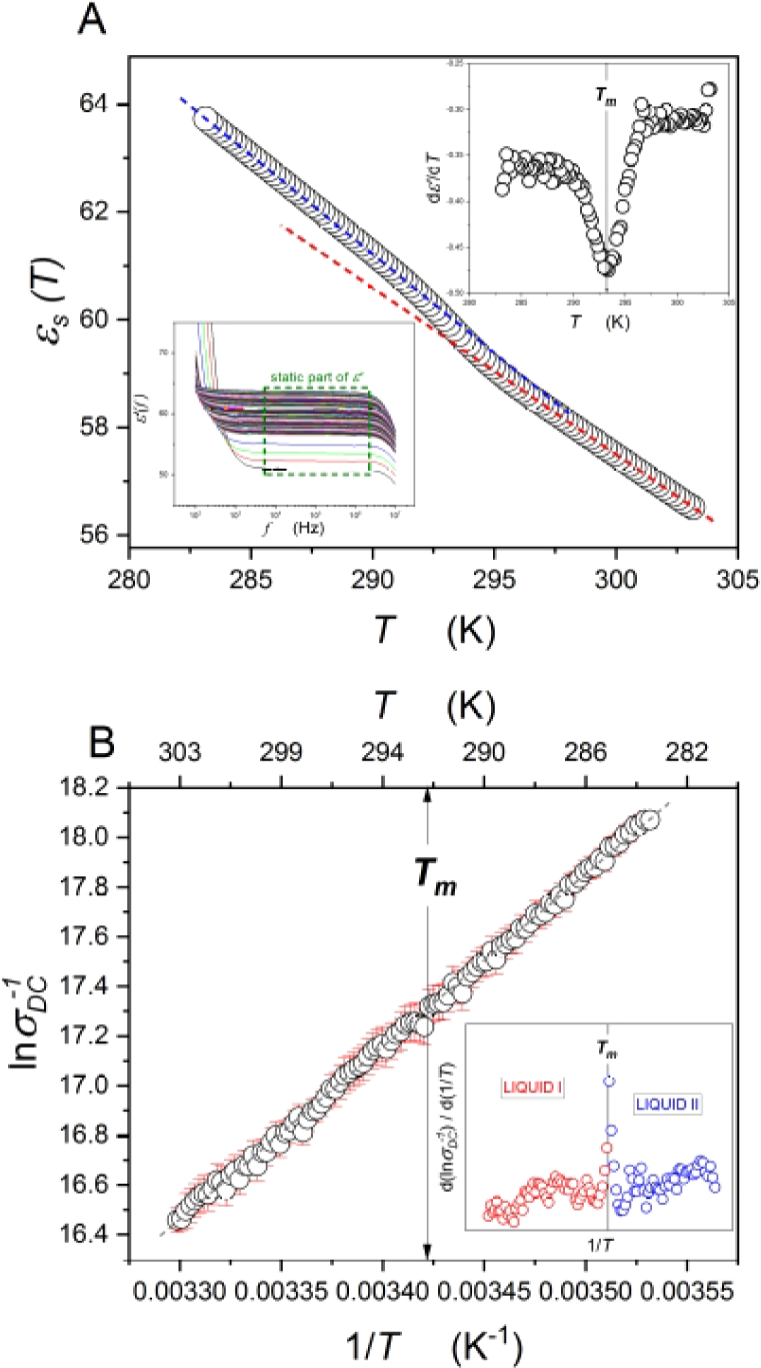
(A) Temperature evolution of the dielectric constant for supercooled glycerol. The left inset presents the frequency-dependent real part of the complex electric permittivity with the marked static region. The right inset shows derivative-based analysis of the dielectric constant *ε*_s_. Characteristic minimum at melting temperature can be found. (B) Reciprocal DC conductivity behavior in vicinity of the temperature *T*_m_. Error bars are marked in red. The inset portrays derivation with strong discontinuity at *T*_m_. The gray straight dashed line presents the typical dependence of DC conductivity in glycerol.

Figure 1 presents the temperature changes of the dielectric constant *ε*_s_ (A) and DC conductivity σ_DC_ (B) in glycerol and its supercooled state. The insets in part (A) show the real part of the complex dielectric permittivity spectra  for all studied temperatures and the first-order derivative of the dielectric constant as a function of temperature , where a minimum refers to the melting temperature *T*_m_. The values of the dielectric constant were taken from the static region, also marked in the data plot. It is worth noting that a characteristic and strong change in the slope of the temperature dependence of the dielectric constant may be observed. Such behavior occurs near the melting point *T*_m_, which is purely apparent for . Another interesting result was obtained from the DC conductivity analysis. Part (B) in Figure 1 shows an anomaly of σ_DC_ around *T*_m_. Derivative-based analysis  showed a strong discontinuity at the melting point. Such behaviors were previously reported for liquid–liquid phase transition.^1,11−13^

This unusual result led us to study nonlinear effects like the nonlinear dielectric effect (NDE) and third-harmonics analysis, which are very useful for analyzing hidden or high-order phase transitions. Moreover, nonlinear methods are very sensitive to critical fluctuations occurring in the vicinity of phase transitions^28−32^ and may be used to define dynamic heterogeneities.^14−19,22,32,33^

**Figure 2:**
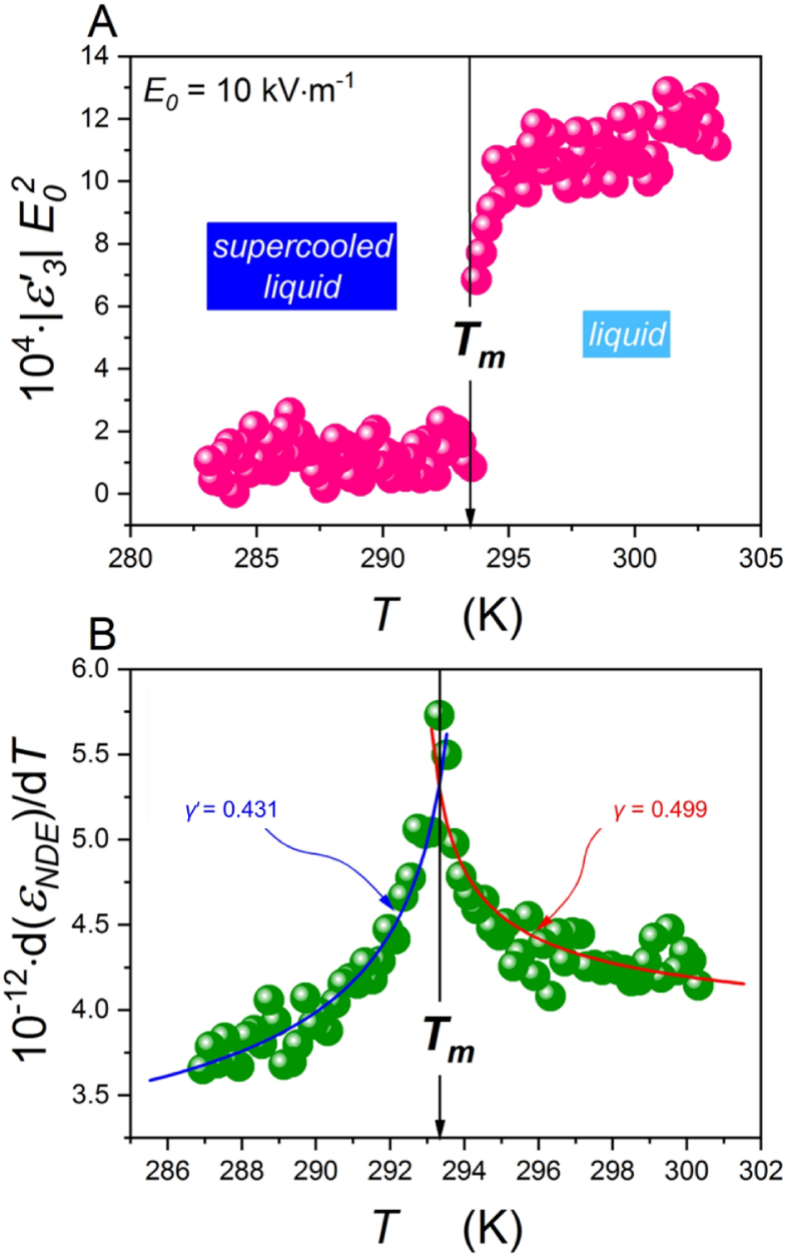
(A) Third harmonic temperature dependence under a weak electric field *E*_0_ = 10 kV m^–1^. Discontinuity can be found at *T*_m_. (B) Derivative analysis of the nonlinear dielectric effect (χ_4_) with critical-like description (eqs 1 and 2) may be found. Blue and red frames contain function parameters. Notably, critical exponents γ = 0.499 and  for  and , respectively.

Figure 2A shows third-harmonic behavior in the vicinity of the melting temperature with a strong discontinuity at *T*_m_, which corresponds to the liquid–liquid phase transition. On the other hand, Figure 2B presents the first-order derivative of the nonlinear dielectric effect in the same temperature regime. The equivalent of the dielectric constant in strong electric fields is the nonlinear dielectric effect (NDE). It is directly sensitive to the appearance of multimolecular heterogeneities/aggregates/fluctuations. The dielectric constant records their average impact on the entire system.

Notably, for *T*_m_, strong critical-like behavior may be observed with characteristic critical exponents: γ = 0.499 for  and  for . More details calculated from eqs 1 and 2 are given in Figure 2B. Such behavior may be connected to the λ-transition, which also can describe liquid–liquid phase transition I metallic glasses.^35^ Based on the above results, we propose to define a liquid above as liquid I, whereas below as liquid II.

According to previous studies,^9,12,13,25^ liquid I corresponds to low-density liquid (LDL), whereas liquid II refers to the high-density one (HDL). It also suggests a type of dynamic behavior, i.e., Arrhenius and non-Arrhenius for LDL and HDL, respectively. Such a consideration is in good agreement with experimental evidence.^9,25,33,34^ The molecular understanding of the observed behavior might be explained by the use of Fischer’s clusters theory^36,37^ also called heterogeneities. For non-aqueous liquids, like “dry” glycerol, molecules can interact by hydrogen bonds, which may produce clusters of molecules. Approaching the melting point *T*_m_, the correlation length of clusters ξ_cl_ increases, forming denser “islands” in a low-density “sea”. The isotropic–nematic, nematic–smectic A, or plastic phase behavior can be used for such a description.^26−28,30,38,39^ High-density “islands” are referred to mostly as heterogeneities or critical fluctuations.^9,12,13,15,30,39−42^ Crossing the critical point, the correlation length becomes large enough to create high-density liquid (HDL) in a supercooled state, which is extremely stable and can be transformed into glassy state.^9,22,23,26−28,30,31^ There is a lack of experimental data taking into account the crystallization process in bulk glycerol. Several results showed such a possibility only in nanopores^40^ or after a very long time of slow cooling.^9,13,17,22^

Liquid–liquid phase transitions between low- and high-density phases represent intriguing phenomena with implications across multiple scientific disciplines. By elucidating the thermodynamic, structural, and mechanistic aspects of these transitions, researchers can advance our understanding of the fundamental properties of materials and pave the way for innovative applications in fields ranging from biology to materials science. Continued interdisciplinary efforts are essential for unraveling the complexities of liquid–liquid phase behavior and addressing outstanding questions in this fascinating area of research. Liquid–liquid phase transitions are intricately linked to the creation of dynamic heterogeneities and critical fluctuations, which arise from the inherent complexity of the system near the critical point. These phenomena play a pivotal role in shaping the phase behavior and dynamics of fluids undergoing phase transitions, highlighting their importance in diverse fields, ranging from soft matter physics to materials science and beyond.

## Conclusions

This report demonstrates that in glycerol, as it approaches *T*_m_, although maintaining liquidity and the absence of a clear liquid–solid transition, changes can be detected through certain physical properties. The report highlights that alterations in the configurations of permanent dipole moments, as evidenced by the dielectric constant and the nonlinear dielectric effect, which is directly responsive to the emergence of multimolecular fluctuations or “heterogeneities” in less ordered or dense environments, can be observed. The findings suggest that the region of supercooled liquids within glass-forming liquids may deviate from the realm of the equilibrium liquid observed above *T*_m_. Notably, there appears to be a discernible indication of a weakly discontinuous liquid–liquid phase transition, closely associated with multiparticle fluctuations.

In summary, our results showed for the first time the shadow of the melting temperature *T*_m_ during the cooling process in glycerol. Analysis of dielectric constant results, as well as comparing them with nonlinear methods, allowed us to define a liquid–liquid phase transition occurring at and covering the first-order phase transition–melting. The presented results also suggest that for *T* > *T*_m_, low-density liquid (LDL) occurs. However, while cooling, strong hydrogen-bonded clusters increased their correlation length, ξ_*cl*_, which produced high-density liquid (HDL). The supercooled state of glycerol is caused by a liquid–liquid phase transition between LDL and HDL, which can be described using critical phenomena. Based on critical-like description, the obtained data suggest a possible λ-type of LL transition with characteristic critical exponents found previously in metallic glasses.^35^ This result may imply that to obtain the supercooled state in H-bonded liquids, the liquid–liquid phase transition is required. Such a statement finds confirmation in many previous considerations.^9,11−13,15,19,21,22^ Understanding the causes and characteristics of liquid–liquid phase transitions continues to pose a challenge, despite mounting evidence of their significance across various systems. This study reveals that such transitions can also occur during the transition to the supercooled liquid state en route to the glass transition temperature. Additionally, it sheds light on novel attributes and underscores the significance of the melting temperature in elucidating the enigma of canonical discontinuous melting/freezing phase transitions.
